# School culture and student mental health: a qualitative study in UK secondary schools

**DOI:** 10.1186/s12889-022-13034-x

**Published:** 2022-03-30

**Authors:** Patricia Jessiman, Judi Kidger, Liam Spencer, Emma Geijer-Simpson, Greta Kaluzeviciute, Anne–Marie Burn, Naomi Leonard, Mark Limmer

**Affiliations:** 1 Bristol Medical School, Population Health Sciences, University of Bristol, Bristol, UK; 2 Faculty of Medical Sciences, Population Health Sciences Institute, Newcastle University, Newcastle upon Tyne, UK; 3 School of Clinical Medicine, Department of Psychiatry, University of Cambridge, Cambridge, UK; 4 Division of Health Research, Faculty of Health and Medicine, Lancaster University, Lancaster, UK

**Keywords:** School culture, School climate, Mental health, Qualitative, Children, Young people

## Abstract

**Background:**

There is consistency of evidence on the link between school culture and student health. A positive school culture has been associated with positive child and youth development, effective risk prevention and health promotion efforts, with extensive evidence for the impact on student mental health. Interventions which focus on socio-cultural elements of school life, and which involve students actively in the process, are increasingly understood to be important for student mental health promotion. This qualitative study was undertaken in three UK secondary schools prior to the implementation of a participative action research study bringing students and staff together to identify changes to school culture that might impact student mental health. The aim was to identify how school culture is conceptualised by students, parents and staff in three UK secondary schools. A secondary aim was to explore which components of school culture were perceived to be most important for student mental health.

**Methods:**

Across three schools, 27 staff and seven parents participated in in-depth interviews, and 28 students participated in four focus groups. The Framework Method of thematic analysis was applied.

**Results:**

Respondents identified elements of school culture that aligned into four dimensions; structure and context, organisational and academic, community, and safety and support. There was strong evidence of the interdependence of the four dimensions in shaping the culture of a school.

**Conclusions:**

School staff who seek to shape and improve school culture as a means of promoting student mental health may have better results if this interdependence is acknowledged, and improvements are addressed across all four dimensions.

**Supplementary Information:**

The online version contains supplementary material available at 10.1186/s12889-022-13034-x.

## Background

Schools are key settings for health promotion, and the concept of a health promoting school has been supported globally [1]. This holistic approach involves not only health education via the curriculum but also having a school environment and ethos that is conducive to health and wellbeing, and by engaging with families and the wider community, recognising the importance of this wider environment in supporting children and young people’s health. There is evidence of positive effects on physical health (including weight, physical activity and diet), and limited evidence for the impact of the health promoting school approach on student mental health [2]. This matters; approximately half of adult mental disorders begin during adolescence [3], making these early years of life a key time at which to intervene to support good mental health, and to prevent or reduce later poor mental health outcomes.

Discreet mental health interventions delivered in schools often focus on improving individual students’ capacity for resilience, empathy, and communication skills and less on school-level factors [4–6]. A systematic review of school-based stress, anxiety and depression interventions in secondary schools found that while those aimed at reducing anxiety and depression were often successful, effect-sizes were mediated by student demographics and dosage, and effects were not long lasting. There was no evidence that interventions targeting stress were effective [7]. The limited impact of discreet mental health interventions may be because they do not address aspects of the school context or system that are determinants of poor mental health, or prevent the intervention becoming embedded [8]. Interventions which focus on socio-cultural elements of school life, and which involve students actively in the process, are increasingly understood to be important for student health and wellbeing [9–12]. Mental health promotion, defined by the World Health Organisation as actions to create an environment that supports mental health [13] is likely to be best achieved in schools that offer a continuum of interventions, including a focus on social and emotional learning, and the active involvement of students [14, 15]. Markham and Aveyard’s theory of health promoting schools proposes that health is rooted in human functioning, which itself is dependent on essential capacities, the most important of which are practical reasoning and affiliation (human interactions and relationships) [16]. These, alongside other (less essential) capacities, make autonomy possible, and allow individuals to maximise their health potential. This is further supported by a systematic review of theories of how the school environment influences health which concludes that for young people to make healthy decisions, they must have autonomy, be able to reason, and form relationships. These capacities are better developed in schools where students are engaged, have good relationships with teachers, and feel a sense of belonging and participation in the school community [17].

The school environment is often termed ‘school culture’ or ‘school climate’; both are used in education literature but neither are well defined and both often encompass many differing and nebulous aspects of the school ethos and environment [12, 18–21]. Some authors use the terms interchangeably; conversely they are also described as separate but overlapping concepts [22]. Van Houtte and Van Maele conclude that ‘climate’ is the broader of the two constructs, encompassing infrastructure, social composition, physical surroundings and culture itself, while ‘culture’ is focused on the shared assumptions, beliefs, norms and values within the school [23]. Rudasill and colleagues propose a Systems View of School Climate (SVSC) as a theoretical framework for school climate research, itself heavily influenced by Ecological Systems Theory [24, 25]. They define school climate as “composed of the affective and cognitive perceptions regarding social interactions, relationships, safety, values, and beliefs held by students, teachers, administrators, and staff within a school.” Wang and Degol reviewed the existing literature and consulted with expert scholars to construct a conceptualization of school climate that includes four dimensions: academic (teaching and learning, leadership, professional development); community (quality of relationships, connectedness, respect for diversity, partnerships); safety (social and emotional safety, physical safety, discipline and order); and institutional environment (environmental adequacy, structural organisation, availability of resources) [21]. In our study, we use the term culture rather than climate deliberately; in a UK context, the term “culture” is far more commonly associated with school environment than “climate” [26]. We use “culture” to capture the broad sense of shared norms, values and relationships specific to each school, and also how student feelings of belonging, safety and support are impacted by the infrastructure and social composition of schools (considered by Van Houtte and Van Maele as part of the broader construct of ‘climate’ [23]).

A positive school culture has been associated with positive child and youth development, effective risk prevention and health promotion efforts, with extensive evidence for the impact on student mental health [23]. Two evidence reviews report strong associations between the student perceptions of the quality of interpersonal relationships within the school, and school safety, and student mental health [18, 21]. School culture may be particularly important to the mental health of Lesbian, Gay, Bisexual and Transgender students who may be more likely to perceive it less positively and be at greater risk of poor mental health, feeling unsafe, and absenteeism [27–30].

Given the evidence base highlighting the importance of school culture and active participation of students in school life on mental health promotion [9, 10, 31], we developed a participatory action research (PAR) approach [32] to understanding and improving school culture in UK secondary schools. Participatory Action Research seeks to enable action within a specific research context by involving study participants as co-researchers. Undertaken in three English secondary schools, our study involves bringing together a small group of students and school staff, facilitated by an external mental health practitioner, to develop a shared understanding of the culture in their own school, and identify changes that might impact student mental health. Participants consider school culture and student mental health, implement changes and/or interventions intended to improve both, and reflect on whether these changes have had an impact. This means that participants are involved in a cycle of data collection, reflection, and action (Act-Observe-Reflect-Plan cycles; [33]. Further information about the PAR study is available elsewhere, including the study protocol [34] and the use of PAR as a research method [34, 35]. At the launch of the PAR intervention, staff and students were asked to reflect on their conceptualisation of school culture in order to develop a shared understanding. Alongside this, the research team undertook qualitative research in each of the intervention schools. Given the differences in the definition and conceptualisation of school culture identified in the literature, we wanted to better understand how it is conceptualised by those most closely impacted by it. The aim of the current study is to identify how school culture in conceptualised by students, parents and staff in three UK secondary schools. A secondary aim is to explore which elements of school culture are perceived to be most important to student mental health.

## Method

This was a qualitative study using semi-structured interviews and focus groups as the primary data collection method. We have followed the Consolidated Criteria for Reporting Qualitative Research (COREQ) checklist [36].

### Research team

The study research team comprised academics from public health centres at four English universities. The development of data collection tools was led by PJ; data collection was led by PJ, LS, and EGT; all of the research team were involved in analysis and reporting.

### Sampling and recruitment

We used a purposeful sampling approach to select schools with variability in school performance (using Ofsted inspection outcomes as a proxy measure for this), and diversity of student intake across ethnicity, and eligibility for free school meals. Three secondary schools were recruited in October 2020, one of which agreed to run two PAR intervention groups. A lead staff contact in each of the schools supported the recruitment of school staff, parents, and students to take part in an interview (adults) or focus group (students), prior to the PAR groups beginning. We worked with this contact to identify school staff with insight into school culture and student mental health and wellbeing. Participants were drawn from the senior management team, teaching staff, other support staff, particularly those with responsibility for student wellbeing (e.g. pastoral support staff, Personal, Social, Health and Economic education (PSHE) lead, head of year, form tutor). For parent participants, we asked for parents with particular insight into the school, for example parent governors, parent volunteers, or those whose children had required extra pastoral support or similar. Potential interviewees were sent a Participant Information Sheet (PIS) that detailed the objectives of the study, interview length and summary of topics covered, recording arrangements, confidentiality, and data protection details, and use of data for reporting. Participation in interviews was voluntary. A consent form was sent to participants by email in advance of an online interview and consent recorded at the start.

In each participating school, all students in the selected year group were invited to take part in the PAR group. School staff shared an information sheet about the PAR group and encouraged students who wanted to take part to contact school staff and also send a short paragraph detailing why they wanted to take part and what skills and attributes they would bring to the group. School staff selected students with guidance and support from the research team (prioritising diversity across gender and ethnicity and those students who were not already involved in any student councils or similar in the school). Students who had volunteered to take part in the PAR intervention, but not selected, were asked if they would participate in the focus group. An information sheet was sent to both students and their carers and consent sought from both to participate (in one school, parents were informed but consent not sought as students were aged 16 years or over). Signed consent forms were collected prior to the focus group and the researchers reaffirmed that consent was informed and voluntarily given verbally at the start of the focus group.

### Data collection

Semi-structured interviews support a structured and flowing interview whilst allowing some flexibility to ensure the respondent can engage with the subject, maintaining more autonomy in how they choose to respond to the topic areas in comparison to a more structured survey method (Adams 2015). Topic guides for the interviews were developed following a rapid review of the research literature on school culture to develop a comprehensive list of components that may impact on student mental health, as well as potential mechanisms through which this may happen (see Additional file 1: Appendix 1). Interviews lasted 30–45 min and guides were used flexibly, using prompts and probes where appropriate. A similar approach was used to develop the topic guide for student focus groups, which included participatory methods to facilitate a discussion about school culture. Focus groups lasted around 45 min.

Data collection took place between December 2020 and April 2021, coinciding with school mitigation measures in place in response to the COVID19 pandemic. These included social distancing, face masks, and year group ‘bubbles’. Although schools were open to all students when data collection began, they were closed to all but vulnerable students and those with key worker parents from the beginning of January until March 2021. As a result, all data collection with school staff and parents took place online. Student focus groups occurred after schools re-opened were a mixture of face to face (3 groups), and online (1 groups) depending on what the school allowed.

### Analysis

All data collection activity was recorded using an encrypted digital recorder and transcribed verbatim. We used the Framework Method of thematic analysis [37, 38]. One of the researchers (PJ) developed a thematic framework after reading several transcripts to familiarise herself with the data and referring to the research questions and topic guide to inform an initial coding stage. This framework was augmented by subthemes that emerged in further transcripts. A short summary of each subtheme was developed to describe the data that it was designed to capture. This initial framework was shared with the whole research team and the thematic framework was further refined until the team were confident that it encompassed all the data in the transcripts, the data within each subtheme was coherent, and that there were clear distinctions between subthemes. The final thematic framework is included in Additional file 2: Appendix 2. PJ then developed a matrix framework, using the subthemes as column headings and participant transcripts as rows. The matrix cells were populated with verbatim and summarised data from the transcripts, as well as analytical notes made by the researchers (‘charting’). Charting reliability was tested by all six researchers charting the same two transcripts independently, and comparing the contents of each cell to ensure that we were applying the subthemes consistently and capturing and summarising the data consistently across all team members. This data management approach produced a data matrix showing data from every respondent under each subtheme, thus providing a detailed and accessible overview of the qualitative dataset. The Framework Method makes possible the capacity to explore the dataset through themes and subthemes, and also by respondent type. A summary of the data under each subtheme was developed to inform the next stage of the analysis, moving up the analytical hierarchy to explore patterns and associations between themes in the data [38, 39].

## Results

### Sample

Information about the sample schools and participants is shown in Table 1.

**Table 1:** Participating schools, staff, parents and students

	School A	School B	School C
School year running PAR group	Y8 and Y10	Y12	Y8
% pupils for whom English is not first language (National average 16.9%)	36	17	21
% pupils eligible for free school meals (last 6 years) (National average 27.7%)	33	20	41
Most recent Ofsted rating	Good	Outstanding	Good
School staff interviews (N)	10	8	9
Parent interviews (N)	3	2	2
Student focus group (N)	2	1	1

Across all three schools, 27 school staff participated in an interview for the study. Staff interviewed included members of the senior leadership teams, teaching staff, learning and support assistants, pastoral support staff, and staff with particular responsibility for the Year group which was taking part in PAR in each school. The parent sample was comprised of seven parents of students in the relevant year groups across the three schools (five mothers, two fathers).

Four student focus groups were held in total; one from each of the year groups participating in PAR across the three schools. Twenty-eight students took part across the four groups; student demographics and online/in school data collection method are outlined in Table 2.

**Table 2:** Student focus group sample

	School A Y8 group (*N* = 8)	School A Y10 group (*N* = 5)	School B Y12 group (*N* = 9)	School C Y8 (*N* = 6)
Gender (self-report)	5F, 3M	3F, 2M	7F, 2M	3F, 3M
Ethnicity (self-report)	1 Black, 2 mixed ethnicity (White and Black British), 3 White British, 1 Somali, 1 Asian British	1 Asian, 2 Black British; 1 White British, 1 mixed ethnicity	2 Black African, 4 White British, 2 White European, 1 Asian other	1 Somali, 1 White European, 2 White British, 2 Asian British
Data collection method	In school	In school	Online	In school

**Fig. 1:**
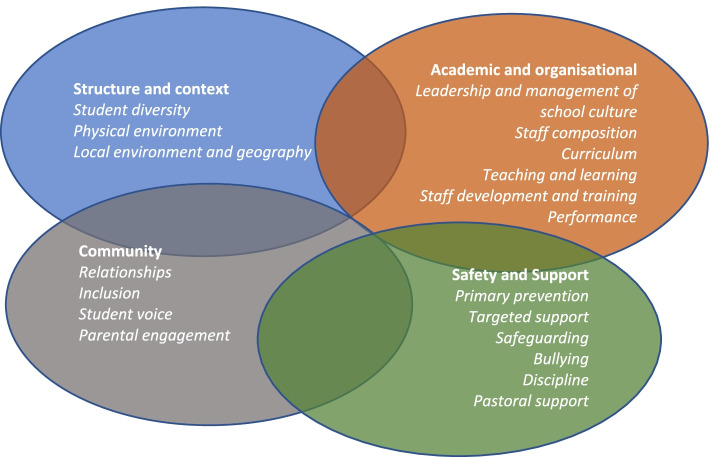
Dimensions of school culture

The findings are presented under four overarching dimensions of school culture that emerged from the data and were perceived by respondents to impact on student mental health. These are structure and context, organisational and academic, community, and safety and support (see Fig. 1). Anonymised quotations are included from a wide range of participants in order to illustrate the responses rather than indicate representativeness. Where differences between participant groups were apparent (e.g. parents, school staff and students) we highlight these in the findings.

### Dimension 1. Structure and context

#### Local environment and geography

School staff noted the impact of geographical location on school culture, with pupils often not living in the immediate locality. This resulted in students living socially deprived areas attending a school in an affluent area, and vice versa. As a result any sense of a school sited within a ‘neighbourhood’ or ‘local community’ setting was depleted. However the impact of the wider locality was recognised. Public events and demonstrations that had occurred in the South-West of England in the 12 months preceding the study generated publicity and awareness amongst the students at all three schools, which staff tried to reflect and respond to.

*“So, being in a city centre, if there’s a protest, it’s on our doorstep. So, the student strikes, on our doorstep, Greta Thunberg, when she came, on our doorstep, Black Lives Matter protests, on our doorstep…[]…So, all these issues, our students are even more exposed to, and, you know, in shaping our culture at school, that’s what we’ve tried to move towards. Not just focussing on inclusivity and care, but also in terms of they’re going to be engaged and informed citizens.”* School B staff 8

#### Student diversity

Almost all respondents referred to the three schools as having a very ethnically diverse student body, bringing both opportunities and challenges. Ethnic diversity was perceived by most respondents as one of the key influences over school culture. Parents often spoke of valuing it as a learning opportunity for their children, and a source of high cultural capital. Many staff shared this view and enjoyed working with such a diverse cohort.

*“The cultural mix at [School A] was really important for me...So culturally, I think the diversity in [School A] is amazing and although it brings with it many challenges, that was a really important thing for me. That my children could see the struggles. I think it is more of a reflection of society…modern society, multicultural society.”* School A parent 1

Many staff noted however that while students may integrate during school hours, they often fell back into homogenous groups at the end of the school day, reflecting the reality of the wider community.

*“This is a diverse school, and the city is sometimes perceived to be a diverse melting pot but it is not, it is still very segregated. There is a lot of work to be done between communities.”* School C staff 5

There was also diversity across the socio-economic status of students. Staff reflected on the severe poverty faced by many of their students, exacerbated during the COVID19 pandemic, and the efforts made to ensure that students were able to access the same educational and wider opportunities as more affluent students. Staff also reported examples of where ethnicity and socio-economic status intersect, impacting the engagement of students and their families with the school.

*“For some BAME families, education is the highest priority. For others who are possibly asylum seekers or who have not really had an education themselves because of issues back home in their own countries, education is much further down the list. You’ve got other families, massive poverty in the families, and so education is the last thing they can think about.”* School C staff 4

Finally staff also noted the influence of students with SEND on school culture. Two schools in particular were perceived to have a high proportion of students with SEND, and staff adapted the curriculum and employed additional support staff to ensure the school environment and offer was inclusive. This included working with all students to promote greater awareness and acceptance of disability.

#### Physical environment

Many staff spoke about the impact of the physical environment of the school on student interactions and wellbeing, and in particular the impact of being quite constrained in a small space. Although efforts were made to create private and safe spaces during break times, often both the number of people, and building and grounds design made it difficult for students to find quiet or perceived safer places to be. This finding emerged in all schools, despite one being an older, traditional building and two being more recently rebuilt to incorporate more light and space.

*“Students do struggle with the building sometimes - a big long tin, built around previous ideas of supervision. So offices are glass, toilets are open, they are non-gendered toilets which are open aside from cubicles, but does mean we are restricted on indoor space - not many places where kids can just sit and relax in social time…So, I think that that’s something they do struggle with.”* School C staff

Students’ capacity to navigate school buildings was further constrained during the pandemic by social distancing measures. Students were often confined to one classroom all day while teachers moved round the school, one-way systems were put in place, and dining areas and school grounds segregated by year group to limit social mixing. Staff perceived this impacted on the school culture, making small incidents amongst students more likely to escalate, and removing teachers’ sense of control in classrooms that no longer felt like their own.

*“All of a sudden they’re crammed, 30 students, into [one] classroom [all day] and I think that’s had a negative influence on a lot of students….[]… As a result small things escalate fairly quickly, which isn’t helping the dynamic within the school. …[]… Before, every time I ever had a class, I would be at the door. I would welcome them into my room, and there’s an automatic element of control and influence where, if there is something, you can address it before you come into the room and the room is the area of control. There isn’t that available anymore, and I thought that that does have an impact.”* School A staff 7

### Dimension 2. Organisational and academic

#### Leadership and management of school culture

The role of the school senior leadership team in shaping school culture was mediated through their support for staff, visibility and transparency to students, and active management of school culture. School staff reported that having a leadership team that listened to and empowered staff was important. This was especially important during the pandemic and related mitigation measures resulting in schools being closed to most pupils and a move to online learning, although for some staff this made the leadership teams less visible. Visibility to students was also seen as key to promoting a welcoming culture in schools; availability and presence during the school day was frequently mentioned by both staff and students.

*“The senior leadership team are very visible to students. They’re out every single lunch and break, every lesson changeover, they’re very hands-on, and I would say that’s probably, I think that’s quite a good sign.”* School A staff respondent 3

The importance of senior staff being present and welcoming students to school each morning was also perceived by student focus group participants as a reason for valuing their school.

Culture emerged as a key priority amongst the leadership team in all three schools, which all take a proactive stance on leading and shaping it, including having senior leaders responsible for it. Stated reasons for prioritising culture included to reflect the needs of a diverse student intake (particularly across ethnicity and socioeconomic status); mitigate the impact of Covid mitigation measures on student wellbeing; and in response to the UK national government’s push for better mental health provision in schools. There was also a sense that newer staff, and staff recently promoted to managerial posts, were more likely to prioritise culture (and student wellbeing).

*“I am aware of how important [mental health in schools] is at the moment from the government.”* School A Parent 1

*“It can be alienating, but they [new leadership team] spoke a lot about culture - something that people say is important, and in general staff are happy…[]...he [Principal] he would start using these quotes from people, and one of the ones that always sticks in my head is ‘culture eats strategy for breakfast’, is one he loved which, again, is on culture.”* School C staff respondent 9

Despite the active management of school culture, there were staff in all schools who questioned whether a narrative of prioritising school culture was tokenistic, without implementing real changes or having noticeable impact.

#### Staff composition

School staff composition was perceived to influence the culture of the school, and mental health of students, through dedicated pastoral and inclusion roles, their ethnic and gender diversity (or lack of), and staff turnover rates.

All three schools had non-teaching staff with roles dedicated to supporting student mental health and wellbeing, including safeguarding (promoting child welfare and protection from harm), pastoral support, mental health support (counsellors), and support and inclusion for pupils with special educational needs and disabilities (SEND). Staff and parents from two schools perceived the wellbeing teams as unusually large compared to other secondary schools. These staff were especially busy monitoring and supporting students during school closures. The importance of staff dedicated to mental health and wider wellbeing support was recognized by all stakeholder groups, including parents.

*“The fact that she [pastoral support lead] doesn’t teach any of them and that they know they can just drop in and they can just go and sit down and say, “I’m having a rubbish day today.” Sometimes that’s what you need. You don’t always need someone to come up with an answer. You just sometimes need somebody to listen.”* School B Parent 1

Beyond dedicated non-teaching staff, many school respondents recognised the role that staff diversity had in shaping and informing school culture. Respondents in all schools were conscious that school staff did not reflect the ethnic diversity of students. Gender representation across teaching subjects and leadership roles was also of concern. There was recognition amongst school teaching staff and leadership teams that students need to see ethnic minority and female role models in all roles, and effort is needed to address this through better recruitment practice.

*“It's the best leadership team I’ve ever worked in [but] If we're talking about representation in there, I am the only non-white person in our leadership team...[]… It's only really in our pastoral teams where we start to see some diversity. That's a real problem in schools that I've always found, is that any kind of black or ethnic minority staff tend to be in the pastoral teams rather than in the teaching and learning teams.”* School C staff 5

Parent respondents highlighted staff turnover and consistency as important. When staff consistency was low, this had a negative impact on students’ wellbeing and school culture as they struggled to build relationships with ever-changing staff. This was particularly important to students who needed additional pastoral and/or inclusion support for SEND or mental health reasons, and also impacted on parents’ ability to build trust and confidence with their child’s key staff contacts in school.

#### Staff development and training

Few respondents mentioned staff development and training as an important aspect of school culture, although some school staff did raise training in specific areas that would influence their capacity to support student mental health and wider wellbeing (including on safeguarding, mental health promotion and prevention, inclusion, and support for students with SEND). Some reported training in new behaviour management policies specifically intended to impact school culture, including restorative justice and holistic approaches. In one school effort had been made to train staff in anti-discriminatory practice, to give them greater confidence in addressing diversity-related issues and supporting students.

*“We need to make sure that, on every bit of our culture that we want to work on, we have staff that are educated in that. I know we've done a lot of work on this year’s staff feeling scared to broach certain subjects, especially with our anti-discriminative practice… They're worried about saying the wrong thing and being accused of being a racist, or being accused of being a homophobe, or being accused of saying something. There's a real fear of that, which I think leads to disengagement, potentially, from trying to be an active participant in the change [to school culture].”* School C staff 5.

#### Curriculum

Respondents across all schools described ongoing changes towards a more inclusive, holistic curriculum, reflective of the diverse student body. The most prominent changes to emerge from interviews were efforts to decolonialise the curriculum across all taught subjects, the inclusion of more content about Black history, and inclusive and diverse content with regard to gender and sexuality. In one school, changes to the curriculum were informed by feedback from student Black and Minority Ethnicity (BAME) and lesbian, gay, bisexual, transgender (LGBTQ +) groups. Staff respondents noted the importance of embedding minority role models across all subject areas, and not simply providing one-off lessons about minority groups. The lack of diversity amongst staff increased the difficulties of delivering a diverse and inclusive curriculum as many reported lacking expert insight, knowledge and confidence. There was consensus however that continuing to work on the curriculum offer was likely to facilitate a more supportive school culture.

*“So, we’re working at the moment unit of work by unit of work by just inputting BAME and female role models and careers. So, that it’s not a tokenistic lesson, it’s actually… it just becomes part of the normal conversation at [School C], and no matter what ethnic group a pupil is from or what their sexuality is, there should be, within the curriculum somewhere, role models popping up…, it becomes part of the day to day conversation.”* School C staff 2

PSHE education was highly valued by both staff and students as an important means of addressing diversity, inclusion, and health. PSHE time was used to deliver universal mental health provision including education, advice, and interventions such as meditation or mindfulness. Students reported that alongside Relationships and Sex Education (RSE), this helped them to develop an understanding of different cultures and to be mindful and respectful of them.

*“I think RSE, PSHE are good because they teach about other people’s cultures and I think it is important since that- say if you don’t know something about another person’s culture you might offend them.”* School C student focus group

For some students, the opportunity to discuss mental health during PHSE was welcomed but could feel tokenistic, without enough time to cover issues in depth, and some stigma around discussing mental health remained.

*“We had a PSHE assembly quite recently and this is going back to the whole 'surface level' thing, because even though the assembly itself was good, it was, like, all the students [in year 12] in one Zoom. So, it was very difficult for us to have actual, proper, discussions. So, it felt quite, "See, we need to have a PSHE lesson at some point, therefore we'll have one big, fat assembly, so we can tick that off our quota," instead of having smaller groups where people can actually discuss their problems and really learn.”* School B student focus group

Staff, parents and students all discussed the importance of non-academic subjects such as Physical Education (PE), Music, Dance and Art, and the ability for students to access these formally through lessons and through lunchtime and after-school clubs. The noted benefits of these include providing an opportunity for self-expression and creativity, a focus on processes rather than outcomes (an important part of mental health), and ‘safe’ spaces to take risks and use failures as opportunities. Staff perceived that having a wider range of music, arts and sports on offer to students allowed them an opportunity to find something they enjoy and may excel in, which may be particularly important for the self-esteem of less academically able students. Respondents across all schools noted that although schools make efforts, there was still not enough emphasis on these wider curriculum areas, and this was exacerbated by the curtailment of after-school and extra-curriculum activities during the pandemic.

#### Teaching and learning

Most respondents’ comments on teaching styles focused on teachers’ attitudes to discipline in the classroom. Where teachers were perceived as overly strict, students and parents worried that this had a detrimental effect on student mental health. Some students and parents perceived that teachers’ focus on academic performance meant they were more likely to overlook student anxiety and stress.

*“For some reason I think [daughter] overthinks things maybe and she second guesses everything she does…[]… They’d set the essay and then she’d spend another week researching which she didn’t need to do. Then she’d start writing the essay by which time other work was coming in, so it got on top of her….[]…She was scared of failing. She was scared of letting them down.”* School B Parent 1

The COVID-19 pandemic may have alleviated this problem and encouraged teaching staff to afford greater consideration of student mental health. Staff were aware that not all students coped well with the closure of schools and move to online teaching and learning for extended periods of time alongside the other stressors of the pandemic.

*“I’ve spent a lot of time on the phone with parents, and sometimes their kids are having a really tough time and they are ditching the distance learning thing. I’m just like, “You know what? Your kid needs to feel better and then we’ll look at the learning again.”* School C staff 3

#### Academic performance

Pressure on schools to be ‘high performing’ was driven both by external regulators and national performance measures (Office for Standards in Education, Children's Services and Skills (Ofsted); Progress 8 scores (progress a pupil makes from the end of primary school to the end of secondary school), and school staff’s ambition to equip individual students with the skills and qualifications for later life. Respondents also noted the impact of schools’ local and historical context; comparisons with other schools in the area (e.g. higher performing schools more attractive to prospective students and parents) and prior status (e.g. as a grammar school, private school or under-performing school) also influenced how the schools’ current academic performance was perceived by parents and staff. All of this influenced how ‘high performance’ was conceptualised. In one school, staff and parents describe the school as highly academic with an expectation that most students would achieve high academic grades and proceed to higher education. In contrast, staff from a school with lower academic outcomes noted that they are driven to maintain year on year improvements in Progress 8 scores, (a ‘value added’ measure) and equip students for a wider range of next steps, including further education, and vocational routes.

*“It is a very high-end sixth form. So you are working with a lot of young people that want to be doctors, vets, quite high-end.”* School B staff 4

*“We were one of the only schools of our kind, really, to see a… I think it for six years, an improvement in our progress 8 scores, which I know are not everything, but actually are quite a good measure for us. We’re an academy that is massively based around progress, and equal progress.”* School C staff 2

Respondents of all types were aware of the impact of high academic expectations on school culture and consequently, student mental health. In school B, which is known for high academic standards, staff reported high levels of anxiety and stress-related disorders amongst students, including eating disorders and self-harming behaviour. School staff acknowledged that expectations of high achievement can cause students stress and anxiety, but addressing this is challenging because it is not always driven by the school culture but by parents or the students themselves.

*“There is a big, big drive for students to apply to Oxford or medicine degrees, dentistry, and in my experience this has caused some significant mental health issues in the students. It’s not necessarily a school issue, I would say it’s more due to the demographics of the students that attend the school. They tend to come from very supportive families, often families that actually want the students to attend Oxford or want the students to be doctors... So there is still this sort of competition with their peers and maybe the frustration of not meeting expectations that come from the parents.”* School B staff 6

### Dimension 3: Community

#### Quality of relationships in school

The quality of interactions and relationships with others in school was perceived as another key element of school culture important to student mental health. Staff respondents distinguished between ‘staff’ and ‘student’ culture, though also recognized that the relationships between staff and students would impact on the school culture overall. Relationships amongst staff were generally described as friendly, supportive and collaborative. This was especially important during the past months when school closures necessitated the move to online teaching, with repercussions for students through better practice, and better support.

*“It’s really noticeable that no matter what department you’re in, what level of teaching, if you’ve got your head around something that other departments or individuals haven’t, people have voluntarily made tutorial videos and just sent them to all staff, it’s not that you have to go knocking and asking all the time, actually people are just pulling together and trying to promote best practice.”* School C staff 2

There was some disagreement over the role of the senior leadership team in reinforcing positive staff culture. Some respondents described working with an empowering leadership team that actively supported good staff relationships. For others, leadership influence over high workloads, pressure to maintain high academic performance, pandemic-related changes and recent staffing decisions (including redundancies) had damaged relationships amongst staff. There was also some stigma around disclosing mental health concerns, particularly those caused or exacerbated by work pressures, although this may be improving.

*“Lots of people are worried about the consequences [of disclosing] and they’re worried about having the label of someone who cannot cope, there’s a lot of that.”* School B staff 6

The quality of interactions between students and staff were perceived as highly influential over how students experienced school culture, and to their mental health. There was recognition amongst school staff that while those in pastoral or other support roles would prioritise maintaining good relationships with students, teaching staff may differ in their perception of their role; some would focus on teaching and learning only, while others would see the creation of positive and trusting relationships with students as important and conducive to better learning and healthy development. Other influences on the quality of relationships between staff and students included pressure on staff and students to maintain high academic standards. Staff willingness to be accessible and approachable to students was also important, and this had been adversely been affected by school closures and subsequent social distancing measures in place in school.

*“Relationships between student and staff are really important, because if you don't really have a good relationship with your teacher, you may feel uncomfortable with asking them for help. It can cause a lot of stress if you're beginning to struggle and you don't get any help.”* School B student focus group

Inclusion and diversity-related factors were key; staff from schools with a more ethnically diverse student intake reported that the lack of diversity amongst staff damaged relationships with students from minority groups. There was also concern that Black and Minority Ethnicity students were over-represented in disciplinary statistics, possibly a result of unconscious bias or prejudice from staff.

*“In our school, when almost all staff are White and then you’ve got an over-representation of Black students in our behaviour data, race becomes an issue. Not just for the students, but for parents as well. That is something we’re continually trying to overcome and work on.”* School A staff 1

Friendships with peers and the quality of interactions between students in school were also recognized as having an important influence on school culture and student mental health by all stakeholders. Respondents across all schools generally described peer relationships as positive, though there was recognition that individual students would have different experiences.

*“If you have good relationships with other students, then your mental health will just, overall, feel better. You'll have someone to talk to, someone to rely on and you'll just, overall, have a better experience at school.”* School B student FG

Diversity, particularly ethnic diversity, was seen as very influential over peer relationships by staff and parent respondents. As noted earlier, it is valued as a key attribute of a school and there is an expectation amongst staff and parents that students will benefit from relationships with peers from different backgrounds. They also report that peer support is strong amongst minority groups, and students with SEND and minority ethnic groups looking out for and supporting each other. However, where problems arise in student relations this is generally attributed to differences across ethnicity, age, gender or disability (with SEND students at particular disadvantage). Respondents describe concerns with discrimination amongst peers in all three schools, which can manifest in a lack of integration during social and break times, and bullying.

#### Inclusion

Efforts to promote inclusion were apparent in all schools, as this was perceived to be another key influence on school culture and student mental health. Across all schools, respondents describe a diverse student intake with regard to ethnicity, socio-economic status, geography, and religion. This was highly valued; forming peer relationships across these divides is seen as an opportunity for students to learn from each other and encourage acceptance and valuing difference. Staff from all schools reported an emphasis on inclusive practices, driven by both the need to ensure that all students felt safe and welcomed in school, and by recent Black Lives matters protests that have highlighted awareness of prejudice and discrimination amongst students.

School staff were conscious that for many students, time in such a diverse environment was limited, and hence the opportunity to gain the most advantage should be optimised.

*“We don't want people to tolerate each other…[]…We want to teach you to celebrate, actually, differences, and learn from each other and be able to have high cultural capital, based on you've got this experience to come to this environment every day where you're mixing with so many different people that maybe, once you leave school, you're not going to be able to access.”* School A staff 5

Staff were keen to emphasise the activities undertaken promote inclusion, such as running groups for under-represented or minority students, (e.g. BAME and LGBTQ + students), increased pastoral support for minority groups, events/displays to celebrate diversity and difference; ‘stamping down’ on issues of intolerance and bullying, and the provision of unisex toilets. There was some recognition of the intersectionality of race, gender and sexuality.

*“Quite often, we find that if you belong to a BAME community, talking about or being open about your sexuality can sometimes be a big no-no…[]…We’ve got a lot of children, students from the BAME community that aren’t out, but actually want to go to the LGBTQ club group to learn and talk and debate and discuss and learn more about themselves. So, you know, making sure they do that in a safe place.”* School C staff 4

School staff perceived some improvements were still to be made, including increasing the ethnic diversity of staff, and addressing potential staff bias that may result in BAME groups being over-represented in disciplinary actions. Staff also report increasing incidences of misogynistic language and bullying amongst students, and this may be the next inclusion issue to be targeted. Students recognise the work that schools are doing around inclusion and value it, although agree that there is still some progress to be made.

*“The school discourse is specifically- I feel like the school used to be a very majority white school and it is slowly integrating and becoming a more culturally diverse school. So, I think the school, in itself, is still learning how to make different cultural identities more heard, more safe, more whatever, but I think the school definitely has a lot more to learn and to do.”* School B student FG

#### Student voice

Student voice and empowerment mechanisms and the success of these varied across the schools and again, were impacted by the pandemic mitigation measures. There was consensus amongst school staff that the degree to which students felt listened to was a key aspect of school culture which would impact on student-staff relationships and student mental health. All schools had systems in place for consultation with and engagement of students (for example student councils). Staff also reported using surveys as a regular means of monitoring student health and wellbeing and gaining feedback on specific issues. There were examples of student-led groups, for example BAME or gender equality groups, being involved in changes to the curriculum or school rules that which particularly affected these groups.

School staff varied in their perception of effectiveness of these mechanisms, with some reporting that school leadership teams were responsive to student feedback and willing to reflect student views. Student councils and surveys had been disrupted during school closures, although respondents across all schools reported that changes had been made to practice (for example, how online learning was delivered), as a result of student feedback. This was a minority view however; the majority of respondents perceived that student views were often ignored and had little tangible impact on the how the school was run. Some attributed this to a lack of staff resource devoted to facilitating and supporting student engagement; conversely other staff report that too much engagement work is staff-led, rather than student-led.

The overwhelming perception of students and parents is that school leadership teams are unwilling to engage with and reflect student views.

*“My personal experience of school councils is that they are a bit of a- we all have a school council because we know it’s the only thing to do but actually when it comes to decision making they kind of ignore it or they’ll steer the kids in a direction they want to go.”* School A parent 1

*'I feel like they try to say that they do a lot [around student voice], especially with student leaders and stuff, but a lot of the ideas and rules that we might want to change get shut down real quick.'* School A student FG

#### Parent engagement

Parental engagement was perceived as good across all three schools. Mechanisms included parent forums, parent teacher association (PTA), email newsletters and social media groups. School staff also liaised with parents of students over specific issues (typically about academic, behavioural, health or SEND support). The degree of communication and engagement with parents increased during the pandemic, as staff conducted additional and regular welfare checks while most students were not attending school in person.

There was recognition that some parents were more willing and easier to engage with than others; factors influencing this include parents’ motivation for their child’s academic success, concern about student support needs, and ‘second generation’ students whose parents also attended the school. The relationship with some parents could be challenging for school staff, either because parents are reluctant to engage, may blame school staff for their child’s behavioural issues or be critical over the perceived lack of support for their child. Engagement was also perceived to vary across socio-demographic status and ethnicity. Some staff reported that higher earning families may have greater expectations of their child’s academic success and will seek out opportunities to engage with school staff to facilitate this. In one school, concerns about problematic disengagement of parents from one particular community was addressed by the employment of a family support worker to liaise between families and schools to help overcome language and cultural barriers.

Most parents were pleased with the level of engagement they had with school staff, particularly where their child had additional needs or existing mental health issues. They report feeling listened to by school staff, who were quick to respond to issues and make necessary changes, making parents feel like they are working together with staff in the child’s best interests.

*“I feel they always know who I am, they know, when I talk about my children they seem to know everything that I’m talking about, and they’re always quick to respond. They actually take you seriously - they sort of think: “Well you’re the parent, you must know your child so tell us what we can do to help.” And I just find that really helpful.”* School A parent 3

Students were aware of parental communication, especially where this was concerned with behaviour or achievement. Many appreciated school staff contacting their parents with positive feedback about them. Students were clear about the links between positive feedback from the school, their parents, and their mental health.

*“Keeping in touch with parents is really important - I would say keeping in touch with parents as when they tell your parents that you’ve been excellent it raises your self-esteem and makes you feel like your parents are proud of you which makes you feel proud of yourself.”* School C student focus group

### Dimension 4: Safety and support

#### Pastoral support

As previously noted, schools all had designated staff for student wellbeing and pastoral support.

Most staff were confident that students would know who to approach, usually a tutor or a member of the pastoral support team. Pastoral staff report being especially busy during school closures, conducting regular welfare checks on all students, and providing additional support for those in need. The pandemic mitigation measures made providing pastoral support harder, by limiting in-person contact while schools were closed, and use of face masks making communication harder.

*“It’s been harder this year because of closures and mask wearing - there’s still a swathe of other [students] that don’t have a connection. You only need a strong connection with one staff member to feel like you’re supported, valued and have someone that you can go to.”* School B staff 8

Parents were universal in their praise for the pastoral support provided to their children, with staff seen as skilled and responsive to both student and parental needs. Students’ views were more mixed, with concerns about anonymity, embarrassment about raising mental health issues in school, unwillingness to approach pastoral staff where they were also teaching staff, and having better support systems in peer groups or at home.

*“There are pretty much only one or two people that I would tell private stuff to, and it definitely isn’t any of the teachers.”* School A Student focus group

#### Primary prevention

Most school staff describe two main mechanisms for mental health promotion; speaking often about the importance of good mental health, and ensuring students in need of support know who to approach for help and guidance at the earliest opportunity. Mental health is addressed during assemblies, as part of the PHSE curriculum. and in tutor time. School staff also used these opportunities to communicate support available to students both within the school from external agencies (via face to face, telephone or online). Teaching staff also have a role in promoting good mental health by having an accessible ‘open door’ policy for students, and being aware of, and not putting further pressure on, students with known mental health issues. Schools also frequently used noticeboards, websites and student newsletters to communicate about mental health.

*“We do things like toilet door campaigns in our school. The inside of toilet doors are just covered in different posters and stuff like that. They’re unisex open-plan toilets. So, we’re targeting everyone with everything”.* School C staff 4

Respondents often commented on the stigma attached to mental health difficulties, and like staff, students may be reluctant to talk about them in school. This was perceived as particularly true for students from some ethnic minority communities. Staff believed that talking often about the important of mental health would encourage students to ask for support if they needed it. Some staff report that as mental health awareness increased, students were becoming more likely to report issues about themselves, or for other students.

*“In terms of preventative support, I want to say the kids really have each other’s back. I would say they really do. There have been lots of cases in the past of friends of students coming to me or going to [Name] or [Name] to say, “So and so is having a panic attack,” or, “So and so is having a tough time.”* School B staff 6

All schools had processes in place to monitor student mental health though the degree to which this was formally structured varied. One had a range of pre-emptive measures in place including regular face-to-face monitoring by safeguarding leads or tutors, and monitoring through proxy measures such as attendance and engagement. School staff also mentioned the importance of staff communication to spot and support students needing support. Student feedback on this was mixed; not all students agreed that it is the role of teaching staff to monitor student mental health.

*“I don't think it’s the teacher’s job to look after people’s mental health. I think their job is just to teach.”* School A student focus group

*“There’s a survey asking you how well do you feel out of one to ten, do you need to talk to someone, how is it going, and all these questions….[]…I think that’s pretty good because then you can just answer it, even though it says your name, no one else is going to see it except the teacher which is fine because they’re the ones who help you.”* School C focus group

#### Targeted support

Targeted mental health support was mainly comprised of access to a school counsellor or a mentor. School respondents often said they would have more targeted support available but this was unaffordable. Staff from two schools also mentioned targeted group interventions for anger management, stress and anxiety, body image, and understanding emotions. Again it was stated than this would be useful for all students as health promotion activities, but the resources were not available. Other barriers to accessing targeted support included pressure on curriculum time; staff reported difficulty in removing a child from a taught class to take part in a mental health intervention. Learning support assistants (LSA) for students with SEND were also perceived to provide high levels of mental health support. Students in all schools were aware of these support systems.

*“Someone that has issues with mental health or wellbeing, they go to their mentor and that would be passed on. Or they could go to the mental health/SEN support teachers for their issues”.* School A focus group.

There were some inequalities in which students are more likely to request, or be offered, targeted support. Staff respondents perceived that BAME groups are under-represented amongst students who access counselling, while white, middle-class students may be more likely to come forward and ask for help with stress or anxiety. There may also be differences in school support provided that are dependent on how the mental health issue manifests; one parent noted that her child may be more likely to receive support because she is ‘likeable and polite’ and hence perceived by school staff as more deserving of support than students with mental health needs that manifest themselves in more challenging behaviours.

School staff also reported signposting or referring students for support from external agencies though waiting lists were often long and may have worsened during the pandemic.

*“Because of funding cuts and things, to actually get referred to CAMHS [Child and Adolescent Mental Health Services]I think you have to meet a very high threshold of maybe being a harm to yourself or others before you get referred. Where, earlier in my teaching career, it was much easier to get support from CAMHS and intervention outside. I think some of it now is just that schools are dealing with so many mental health issues as teachers and staff that, maybe at other times, might have been dissipated to other organisations and things like that. I guess that’s been exacerbated in COVID maybe.”* School B Staff 1

Parents mostly reported satisfaction with the level of support put in place by schools for their children, many of whom had required targeted mental health support. For some, there was a preference to manage mental health support within the school where possible to benefit from existing trusted relationships, even if this meant some delay.

*“I did ask for him to be referred [to the counsellor], but what was interesting is there’s a very, very long list of people. …[]… I’ll see how long it takes, because I will seek it in a different way, privately, if necessary. But I think again there’s a sense of safety. I think [Son] does trust the school, I’m not saying he likes all the teachers or anything, because he doesn’t, but he trusts the school. So, I figured if it came through the school, it’s joined up; he would feel safer.”* School C parent 1

#### Safeguarding

Safeguarding was prioritised by staff, but did not emerge as a prominent element of school culture. All schools had designated safeguarding leads and protocols in place. There were some differences between school staff and students in the understanding around safeguarding protocols. Some believed that students understood the importance of safeguarding, and if disclosures (of risk or harm) are made, then staff had a duty of care to act upon them. However this had the potential to damage trust and limit how much students were willing to share with school staff.

*“The school counsellors have a reputation of saying it’s confidential but then still telling your parents and stuff.”*

*“Some people just want to talk to the teacher without having any consequences, and they’re not in any danger, but then they feel like their information will be [shared].”* School A student focus group

#### Bullying

For many school staff, issues with bullying were closely linked with diversity and inclusion. Misogyny, and prejudice against students with SEND were perceived to be the more pressing underlying causes of bullying. As such, many anti-bullying initiatives in schools were also inclusion initiatives, such as support groups for minority and vulnerable students, addressing inclusion and diversity in the curriculum, and celebrating diversity during tutor time and assemblies. Students often perceived these approaches to bullying as too simplistic, and not addressing the more coercive types of behaviour they experienced.

*“I think there’s quite a lot of assemblies and stuff, but I think it’s not always presented in the right … it’s like very stereotypical bullying… rather than there’s lots of different types of bullying and sometimes not all of it is covered, like manipulative people and people who try and get you to do stuff, but that’s not [discussed]. Whereas that is actually bullying if they’re trying to get you to do stuff….[]…sometimes if you’re being targeted, you don’t realise that they’re bullying you because it hasn’t been shown in anything and you haven’t seen that as bullying.”* School A student focus group

Staff described anti-bullying messages displayed around the school, and students were taught about bystander apathy and to challenge bullying behaviour. One school had anonymous reporting for students to report bullying. For many students however, recognizing and reporting bullying to school staff remained problematic.

*“I feel like sometimes yes, we go and talk to the teacher about it but sometimes some students might feel that peer pressure into not doing it …[]… because they’re like, “Don’t do this or I’ll do this to you.”* School C Student focus group

#### Discipline

Staff respondents discussed changes in disciplinary procedures over recent years, with a shift away from punitive disciplinary systems which appeared to no longer work (or have an even more negative outcome on existing behavioural issues) towards restorative approaches and building relationships/rapport between staff and students.

As noted, some staff reported concerns that BAME students were more likely to be subject to disciplinary measures, which may be due to real differences in behaviour or, conscious or unconscious bias amongst (predominantly) white staff. Two schools had adopted systems emphasising ‘rewards before sanctions’; students could win points through positive behaviours. Students with challenging and disruptive behaviours at risk of sanctions were also offered additional contact, monitoring and support from staff, to promote positive relationships. Staff reported some implementation issues, with staff training on restorative justice approaches disrupted by the pandemic, and some inconsistency in how discipline was applied. Some staff perceived that students themselves have a key role to play in maintaining discipline and modelling good behaviour.

*“If a student doesn't hold open a door for someone else, that is something you pull them up on. Any kind of bad language you hear, whether it is in a classroom or whoever it is directed at, is challenged. Uniform is absolutely 100%. Every single member of staff challenges it. So then the students become role models for anyone coming in because I think, particularly teenagers, although there are lots of exceptions to this, but generally speaking they don't really want to stick out too much. They don't want to be the one who is doing the role or a different thing from their peers.”* School B staff 2

## Discussion

There are many examples and conceptualisations of school culture in the literature emerging from reviews of school culture research, and its measurement [12, 20, 21]. The aim of this study was to identify how school culture is conceptualised by staff, students and their parents in three UK secondary schools. It adds to the literature by providing a conceptualisation that is grounded in the experience of those within UK secondary schools: staff, students, and their parents.

Respondents from three schools identified elements of school culture that align into four dimensions; structure and context, organisational and academic, community, and safety and support. Structure and context includes physical aspects of the school buildings, the geographical setting, and the diversity of these on the student intake, particularly around ethnicity and socio-economic status. The academic and organisational dimension includes how culture is led and prioritised by school leaders, pedagogical aspects including teaching and learning styles and the curriculum, academic performance, and staff composition*.* Community refers to the quality of the relationships within and across key stakeholders in any school; students, parents (or carers), and school staff. Safety and support primarily refers to how schools support student emotional and psychological wellbeing, including through the provision of both primary and targeted support for mental health, although some aspects of physical safety (for example, bullying) may also be important.

A secondary aim was to explore which elements of school culture are perceived to be most important to student mental health. While elements across all four dimensions have influence, respondents were most likely to discuss diversity (across ethnicity, socio-economic status, gender and sexuality) in both the student and staff population as a key element of school culture likely to influence student mental health. This is supported by a recent study of over 28,000 adolescents in England which found gender, ethnicity and deprivation were risk factors for experiencing mental health difficulties [40]. Other elements of school culture that emerged as key influencers of student mental health were inclusive practice as an important element of mental health promotion, pastoral support, the quality of relationships and interactions in the school, and student voice, although mechanisms to promote student voice were regarded as unsatisfactory by most respondents, particularly students.

This study also demonstrates how culture was prioritised by staff in the participating schools. Senior leaders recognised the importance of culture and took a proactive stance on leading and shaping it. This was driven by their belief that it will influence student mental health, and the UK Government’s emphasis on the role of schools in supporting mental health [41]. It is also apparent in our data how school staff were influenced by wider events, including the COVID19 pandemic and the subsequent impact on mental health, and the Black Lives Matters protests of 2020. School leaders (and all school staff) reflected on the impact of these events on student mental health and the need to respond and adapt aspects of school culture in response.

The four dimensions identified in our study closely align with those identified in Wang and Degol’s conceptualisation of school climate [21], though there are some differences. Their ‘institutional environment’, referring to the physical school building and allocation of resources, is replaced in our study with ‘structure and context’. Participants in our study placed little emphasis on the quality of the physical environment (school buildings, maintenance, cleanliness etc.) although building design did feature. Instead, this dimension included greater emphasis on contextual factors including the school’s geographical setting and the diversity of the student cohort. In particular, stakeholders in our study perceived that the ethnicity, socio-economic status and to a lesser extent, intellectual disability (SEND) characteristics of the student intake had a profound effect on the culture of the school and staff efforts to manage it. Unlike other models of school culture (or climate), which consider the social composition of the student body as outside the construct of school culture but hugely influential over it [23, 24], in our study the social demographics of the student intake was one of the defining features both of the school culture and of efforts to manage and improve it.

How might the dimensions of school culture as conceptualised in our study influence student mental health promotion? Wang and Degol’s dimensions of community (in particular, the quality of interpersonal relationships within the school) and safety, which largely overlap with those dimensions in our study, were found to be key determinants of students’ emotional wellbeing [21]. Much less research has been undertaken on the impact of academic and organisational factors on psychological outcomes (though more has been done on their influence over academic outcomes). Markham and Aveyard’s theory of health promoting schools suggests that schools can promote (or inhibit) the capacities essential for human functioning, and therefore health, through ‘framing’ and ‘classification’ [16]. ‘Framing’ refers to pedagogic practice, and ‘weak’ framing is that which enhances student involvement in their own learning and opportunity to influence the curriculum, thus increasing capacity for practical reasoning. This is reflected in our study through respondents’ accounts of curriculum development and efforts to enhance student voice and engagement. ‘Classification’ refers to the boundaries between students, their peers, school staff, and the outside world. Drawing on Bernstein’s theory of cultural transmission [42], the authors advocate that strong boundaries ‘insulate’ students and prevent opportunities for both forming relationships (affiliation) and practical reasoning, the two capacities most essential to mental health optimisation. In our study, efforts to promote better relationships between peers and with staff, engage with parents and develop the curriculum to better reflect the wider world and incorporate diversity could be interpreted as efforts to weaken these boundaries and hence promote affiliation.

Studies which focus on the influence of one aspect of school culture on student mental health are useful, but may miss the wider effects of school culture. The boundaries between the four dimensions identified in our study are not distinct, but factors within each one have influence across all dimensions. Diversity of the student intake, in particular ethnicity, is a key factor in the structure and context dimension but also hugely influential over factors in the other three dimensions. It was particularly salient to our respondents when describing delivering and adapting the curriculum (including efforts to decolonialize it), and their concerns about staff composition. Ethnicity also influences community factors; lack of minority representation amongst staff is seen to damage relationships with BAME students, and drives the emphasis on inclusive practice evident in all three schools. Staff were also cognisant of the influence of ethnicity on disciplinary practice, and student perception of the equity of this. Another illustration of influence across dimensional boundaries is how efforts to create a safe and supportive environment influence factors within the academic and organisational domain, such as staff training on inclusive practice and the inclusion of mental health in the curriculum. This study makes clear the interdependence of the four dimensions in shaping the culture of a school. School staff who seek to shape and improve school culture as a means of promoting student mental health may have better results if this interdependence is acknowledged, and improvements are addressed across all four dimensions rather than prioritising one or two.

A strength of this study is the inclusion of the student voice, and of students across a range of ages and ethnicities. This is unusual in school culture literature. However we are limited in the generalisability of our findings given that participants were drawn from only three schools, from one geographical area, during a global health emergency. We are also limited by the selection of parents for this study, as gatekeeping effects of school staff may have resulted in the exclusion of parents with a more critical appraisal of the schools. Further work is required to determine if this conceptual model of school culture is transferable to other schools in different contexts (with within the UK and beyond).

As the PAR intervention is implemented in our study schools, we plan further research with school-based participants to explore how the active involvement of students as co-researchers working to improve school culture for the benefit of student mental health works in practice. This methodology reflects the importance identified in the literature of active engagement and the promotion of autonomy in health promoting schools [9, 14, 16, 17]. Studies are also needed that identify effective ways in which to influence all the different dimensions of school culture, to ensure safe and inclusive environments that are supportive of and not detrimental to student mental health.

## Supplementary Information

**Additional file 1.** Appendix 1

**Additional file 2.** Appendix 2

## Data Availability

The data that support the findings of this study, including interview and focus group transcripts and other research materials including participant information sheets have been made available on the data depository data.bris.ac.uk. Restrictions apply to the availability of these data, and researchers may gain access on reasonable request from the data.bris after their host institution has signed a Data Access Agreement. https://doi.org/10.5523/bris.2k2vyu5s93ztd2dk4fhg8ukqqk, https://data.bris.ac.uk/data/dataset/2k2vyu5s93ztd2dk4fhg8ukqqk
