# Supplementary material for: School culture and student mental health: a qualitative study in UK secondary schools
Source: BMC Public Health. 2022 Mar 30;22:619. doi: 10.1186/s12889-022-13034-x (PMC8964383; doi:10.1186/s12889-022-13034-x)
Supplement: Supplementary file 1 — Additional file 1. Appendix 1 [file 12889_2022_13034_MOESM1_ESM.docx]

# **9. Topic Guide for school staff (pre- and post- PAR activity in the school)**

*RQs of relevance to this topic guide:*

***1: What are the key components of school culture that impact on student mental health and how can they be measured?***

***2 What changes or interventions can be made to improve school culture and mental health outcomes for students?***

***3. What is the process by which school culture impacts on young people’s mental health?***

***5. (Post-PAR interview only) Is a Participatory Action Research approach feasible and effective as a methodology for instigating positive change to school culture?***

*Note: This topic guide is indicative and will be subject to incremental change as new findings/information emerge during the research. It is a guide to the topics to be covered during the interviews (and not a script) therefore the order of topics, and the precise manner in which they are addressed, will be flexible according to the circumstances.*

**Introduction:**

- Thank you for participating
- Introduce self and NIHR SPHR
- Introduce the study
- Talk through key points:
- length of interview
- interview like a discussion, but will cover key topics
- no right or wrong answers
- participation is voluntary, rights to withdraw
- recording interview (concentrate on what you are saying, accuracy)
- Confidentiality and anonymity, how findings will be reported
- Questions?
- Happy to proceed? Sign consent form

1. **Participant background/role**

- Clarify current role and responsibilities
- Length of time at current school and previous experience
- If appropriate, role and responsibilities in relation to child wellbeing and mental health

2. **Setting the context – the school (pre-PAR interviews only)**

- School type – academy, part of MAT, LA-maintained
- Size
- Leadership- structure of senior leadership team
- Influence/involvement of governors/parents
- Demographics of local area/school students
- Academic performance and Ofsted rating
- Impact of all of this on school culture
- How is school culture prioritised (or not) in this school and what influences this?
- Does your school have any policies that relate to school climate?

**3. School culture**

- How would respondent describe the culture in the school
  - For staff, as a place of work
  - For students
  - For parents
- Any recent changes (why)
- is there anything you have done differently last term or during this current lockdown that has created a better school culture?
- What in their view are the main influencing factors on school culture
- Any activities/interventions to influence school culture
  - Drivers for these
  - Success/impact

**4. School culture – logic model**

- What would you say are the key components of school culture?
- *Other Components of school culture* (for each one, respondent will consider whether they agree this is a key component of school culture; their perception of this factor in their own school (policies/interventions to support this/whether it is working well/contributing to a positive school culture or not/could be improved). Components to include:
  - School discipline, and fairness of application
  - School rules and norms
  - Availability of caring adults
  - School leadership and staffing
  - School belonging
  - Physical environment that supports wellbeing
  - Safety
  - Availability of targeted support for students at risk of poor mental health
  - Support for minority groups (race, gender, sexual identity, disability, socioeconomic, and/or cultural differences) – inclusion and support
  - Anti-bullying initiatives (includes online)
  - Relationships
    - Between staff
    - Staff-student
    - Student-peer
    - Staff-parent
  - Student involvement
  - Teaching and learning styles
  - Social and emotional earning (PHSE; RSE)
  - Parent involvement
  - Civic/community activities
  - Policies
- Are there other important components of school culture missing?
- Which components would the respondent see as essential for (rather than simply contributing to) a positive school culture?
- Now consider the *potential impact* of a positive school culture on student mental health. Respondent to comment on each if they perceive school culture can influence this outcome; if it does in their school; how these outcomes might be demonstrated/measured/perceived. Outcomes on student mental health to include:
  - Feeling physically safe
  - Feeling socially/emotionally supported
  - Self-esteem
  - Positive and supportive relationships
  - Challenging behaviours
  - Risk behaviours
  - anger/aggression
  - bullying/victimisation
  - mood
  - motivation to learn/engage
- And non-mental health outcomes may include
  - Absenteeism rates
  - Exclusion rates
  - Academic performance
- Are there any students outcomes missing from this list?

**5. Mechanisms through which school culture may impact on student mental health**

For example, improving school culture may:

- Encourage values of inclusivity, equality, empowerment and respect
- Create an environment in which creativity and innovation are valued
- Reduce factors that contribute to poor mental health (stress, bullying etc)

For each one:

- Does the respondent agree/disagree that this is how student mental health may be influenced? Is this realistic?
- Is this what happens/might happen in your school?
- What are the facilitators for change (those factors support having a positive impact on student mental health)

**6. PAR approach (questions in the pre-PAR interviews only)**

If necessary, interviewer to explain the PAR groups and how they will operate in school; goals and objectives.

- How does the school currently involve young people in decision-making?
- What are your perceptions of the PAR approach to school culture?
  - Advantages of method
  - Disadvantages of method
- What are your aspirations for the PAR group?
  - Outcomes for students involved
  - Outcomes for staff involved
  - Outcomes for school as a whole
- What ideas/initiatives do you think the PAR group might suggest?
  - If different, what activities/initiatives *should* they suggest?
- What needs t be in place to support the PAR group having a positive influence on school culture and student wellbeing?
- What are the potential barriers and how might these be addressed?
- How might the impact of the PAR group on school culture/student wellbeing be measured?

**7. PAR approach (questions in the post-PAR interviews only)**

- How aware are you of the work of the PAR group in your school over the last year?
  - Why is this
- What initiatives did the PAR group suggest?
  - Were any initiatives refused/declined? Why?
- Of those that were implemented
  - What were these?
  - Why do you think they were chosen?
  - How successful or otherwise was the implementation?
    - Barriers/facilitators to this
  - What was the impact of the initiative (s)
    - On school culture
    - On staff/student wellbeing
    - Other outcomes (incl parents)
  - How do you know?
    - Perception
    - Evidence gathered
- If there were positive outcomes for student mental health, were these
  - Experienced by all students
  - Differentiated by age, socio-economic status, ethnicity, academic performance
  - With this in mind, do you think the PAR approach has impacted on inequality in mental health outcomes for students in your school?
- What are your perceptions of the PAR approach to school culture?
  - Advantages of method
  - Disadvantages of method
- What do you think the impact was for members of the PAR group?
  - Outcomes for students involved
  - Outcomes for staff involved
- If your school was to run the PAR group again, what changes would you make?
- How feasible is it for other secondary schools to implement the PAR approach to school culture improvement?
  - Resource (staff time/money)
  - Motivation/priority
  - Impact on student time/curriculum
- What needs to be in place to support a PAR group in having positive influence on school culture and student wellbeing?
- What are the potential barriers and how might these be addressed?
- How might the impact of the PAR group on school culture/student wellbeing be measured?

**7. thank and close**

- ask if anything else they'd like to add
- any key documentation that would be useful to share with study team
- thank and close.
