# Supplementary material for: School culture and student mental health: a qualitative study in UK secondary schools
Source: BMC Public Health. 2022 Mar 30;22:619. doi: 10.1186/s12889-022-13034-x (PMC8964383; doi:10.1186/s12889-022-13034-x)
Supplement: Supplementary file 2 — Additional file 2. Appendix 2 [file 12889_2022_13034_MOESM2_ESM.docx]

**Thematic Framework for PAR interviews**

**Central Chart**

*i. School* (A, B, C)

*ii. Respondent Type* (School staff, Parent, student)

**Thematic framework**

**1. Background information**

*1.01 Role* - will include job title and description of role for staff; hours worked, parent and age/school year of child(ren) for parents and any other school-related responsibilities e.g. governor. Include gender of participant. For parent respondents, details of family, experience of MH issues to be logged here.

*1.02 Experience* – will include length of time in role, any previous experience/responsibilities. For parents may include other aspects of life that give insight e.g. own profession

*1.03 MH role –* primarily for staff, any specific details of role that include responsibilities for student wellbeing and mental health. Also include any specific safeguarding responsibilities here.

*1.04 Other*

**2. School context and culture** (information about the school that the staff member/parent is attached to. Includes factual information and also perceptions of how this influences the culture at the school)

*2.01 Structural information* - school type e.g. MAT, size, teacher/student ratio; religious school

*2.02 Wider contextual factors* – geography, setting, history of the school.

*2.03 Demographics* - of students, staff, local area and influence of this. Includes gender, ethnicity, SEND, pupil premium, Looked-after, children in need etc. Any details on proportion of students with MH/wellbeing support needs.

*2.04 Leadership* - structure of school leadership team, leadership style/ethos, (recent) changes in leadership. Includes any details of school governors. Influence of this.

*2.05 Staffing:* any information on school staffing beyond leadership. New roles/changes in structures, particularly around pastoral/inclusion/support staff. How pastoral and support staff are managed/team structure. Staff turnover. Links between non-teaching (e.g. pastroral/support) and teaching staff. Influence of this on school culture. Include planned changes.

*2.06 School performance* - academic performance, Ofsted, other indicators of school performance. Influence of this. Academic aspirations/expectations on students (and impact on these on wellbeing)

*2.07. improvement/training* – any content re school improvement processes, staff training, CPD. Impact on culture/student wellbeing. Impact on staff. Drivers and outcomes.

*2.08 Parental involvement* - in governance; connectedness; general communications with parents; involvement in student learning/support etc. Success/otherwise of this and reasons.

*2.09 Managing school culture* – is creating a supporting culture a priority of the school, including staff/student wellbeing how does this manifest (policy, strategy, practice); reasons.

*2.10 – perception of school culture –* any definition of school culture provided. Any data on how current school culture is perceived by students, staff, parents. How they perceive the culture, and why.

*2.11 changes to culture* – any recent changes to culture (prioritisation, perceptions etc) and why. Include any initiatives to influence school culture, drivers for these and success/otherwise

*2.12 COVID19* measures *and impact* - anything done differently as a result of pandemic restrictions that has impacted school culture. Positive and negative changes. Things they will keep post-pandemic. impact on student wellbeing/MH; staff; culture

*2.13 Other*

**3. Logic Model and Culture components**

3.01 *Discipline –*behaviour management, fairness of application. Also any overarching strategies e.g. 3 strikes and out; restorative justice models etc. Include praise/rewards systems, positive behaviours. Include planned changes/interventions.

*3.02 School rules and norms –* other rules/norms mentioned outside behaviour management e.g. uniforms, mobile phone use etc. Regular celebrations, reward assemblies, coffee and cake times etc Include planned changes/interventions.

*3.03 Pastoral support –* pastoral staffing, inclusion teams, other support staff. Role or all school staff. Student awareness of pastoral/MH/Wellbeing support. Availability of caring adults. Include here any details or examples of staff members who are particularly important/play a key role.

*3.04 School belonging* – do students have this/sense of connectedness to school. Reasons why (not). Anything done to encourage this.

*3.05 Physical environment* – aspects of school buildings/grounds that support/impede student wellbeing/positive culture. This may include physical restrictions currently/previously implemented due to COVID19 . Include planned changes/interventions.

*3.06 Safety –* how safe do students feel, and reasons. Initiatives/policy around this.

*3.07 Primary prevention* – promoting good mental health e.g. initiatives, interventions, includes eg peer mentoring, themed assemblies etc

*3.08 Targeted support MH* - Availability of targeted support for students known to have, or at risk of, poor mental health. Any information re liaison with external agencies such as CAHMS. Include planned changes/interventions. Differences between students who require this (e.g. by age, gender) and or differences in help-seeking behaviour.

*3.09 Monitoring student MH –* how potential MH issues are identified and monitored. Include monitoring or recording systems, meetings, which staff are informed

*3.10 Inclusion* - inclusion/diversity issues - race, gender, sexual identity, disability, socioeconomic, and/or cultural differences) – inclusion and support. Any support targeted towards minority groups. Any initiatives to promote inclusion. Include planned changes/interventions.

*3.11 Safeguarding:* How cases dealt with. Staff involved. Procedures. Student/parent involvement. Include planned changes/interventions.

*3.12 Anti-bullying initiatives* – includes online and social media; on and off site. Include planned changes/interventions.

*3.13 Relationships* – all relationships e.g staff/staff; staff/student; peers; staff/parents etc. This includes peer support between students.

*3.14 Student involvement* – student voice, councils, representation. How these work, have influence (or not), representative of student body. Include planned changes/interventions.

*3.15 Teaching and learning styles* – any initiatives/strategy around this. Inclusion of those with SEND. Include planned changes/interventions. Use of sets/streams.

*3.16 Curriculum –* In particular social and emotional earning (PHSE; RSE); Initiatives such as decolonialisation. Includes after-school activities

*3.17 Civic/community activities* - student activity in community coordinated by school – fundraising, projects. Community involvement in school e.g. in teaching/learning. Include afterschool activity. Include planned changes/interventions.

*3.18 Breaks –* issues around break/lunch/afterschool times. Level of supervision. Where students can go, how they are allowed to spend their time. Impact on MH/wellbeing/culture

*3.19 Policies –* any policy/strategy specifically intended to influence/create a positive school culture school culture; staff awareness and perception of these

*3.20 other culture components –* mentioned by respondents but not any of the above sub-themes

*3.21 wider influences on MH* – non-school factors influencing student MH e.g. family circumstance, social media, national policies, societal influences etc.

*3.22 key influencers* - What in their view are the main influencing factors on school culture

*3.23 impact on mental health* – perceptions of influence of school culture on student MH outcomes. includes outcome such as feeling physically safe, socially/emotionally supported, self-esteem, having positive and supportive relationships, challenging or risk behaviours, bullying/victimisation, mood. Any other MH outcomes

*3.24 impact on other student outcomes* – e.g. absenteeism, exclusion, academic performance

*3.25 mechanisms* - potential mechanisms through which school culture may impact on mental health. E.g. Encourage values of inclusivity, equality, empowerment and respect/Create an environment in which creativity and innovation are valued/Reduce factors that contribute to poor mental health (stress, bullying etc). content relating to these and other mechanisms to be included here, including whether they see this in own school.

*3.26 barriers to change -* barriers to influencing school culture and student mental health. May include factors such as resources, staff time, pressure on academic outcomes/school performance, influences on mental health outside the school, prioritising culture/mental health

*3.27 measurement* – any content relating to measurement/observable changes in student outcomes as a result of school culture – how would we know?

*3.28 Other*
